# Optimization of the X-ray incidence angle in photoelectron spectrometers

**DOI:** 10.1107/S0909049513007747

**Published:** 2013-05-01

**Authors:** Vladimir N. Strocov

**Affiliations:** a Swiss Light Source, Paul Scherrer Institute, CH-5232 Villigen-PSI, Switzerland

**Keywords:** photoemission, X-ray absorption, photoelectron attenuation, photoelectron spectrometers

## Abstract

The interplay between the angle-dependent X-ray reflectivity, X-ray absorption and the photoelectron attenuation length in the photoelectron emission process determines the optimal X-ray incidence angle that maximizes the photoelectron signal. Calculations in the wide VUV to the hard X-ray energy range show that the optimal angle becomes more grazing with increasing energy, from a few tens of degrees at 50 eV to about one degree at 3.5 keV. This is accompanied by an intensity gain of a few tens of times, as long as the X-ray footprint on the sample stays within the analyzer field of view. This trend is fairly material-independent. The obtained results bear immediate implications for the design of (synchrotron-based) photoelectron spectrometers.

## Abstract 2

The interplay between X-ray reflectivity, X-ray absorption and photoelectron attenuation in the photoelectron emission process is analyzed. The optimal X-ray incidence angle to maximize the photoelectron signal is evaluated in a wide VUV to hard X-ray energy range.

## 1. Introduction   

X-ray photoelectron spectroscopy (XPS) experiments are in general characterized by a disparity of some two orders of magnitude between the relatively large X-ray attenuation depth and relatively small photoelectron escape depth. Most of the photoelectrons are excited therefore at a depth much larger they can elastically escape from, and on their way to vacuum dissipate in a series of inelastic scattering events to form the secondary-electron cascade background that carries little spectroscopic information. Obviously the way to gain the elastic signal will be to deposit more of the X-ray energy closer to the surface, which can be achieved by using grazing-incidence angles.

The process of the X-ray-excited production of photoelectrons was cast into an exact numerical framework in 1972 in the seminal work of Henke (1972 ▶). Henke showed that a significant increase of the elastic (no-loss) photoelectron yield can be achieved with grazing X-ray incidence angles approaching the total external reflection (critical) angle α_c_. These results have received further theoretical developments (Fadley, 1974 ▶) including generalization to multilayer structures (Chester & Jach, 1993 ▶; Fadley *et al.*, 2003 ▶) as well as extensive experimental verification (Hayashi *et al.*, 1996 ▶; Kawai *et al.*, 1995 ▶).

Here we analyze the interplay between the X-ray reflectivity, X-ray absorption and photoelectron escape processes with particular attention given to the effects of the photon spot size with respect to the analyzer field of view (FOV). The optimal X-ray incidence angle to achieve the maximal XPS intensity gain is determined in a wide energy range from VUV to hard X-rays. These results bear immediate implications for optimization of the experimental geometry of (angle-resolving) XPS spectrometers.

## 2. Formalism   

**Figure 1:**
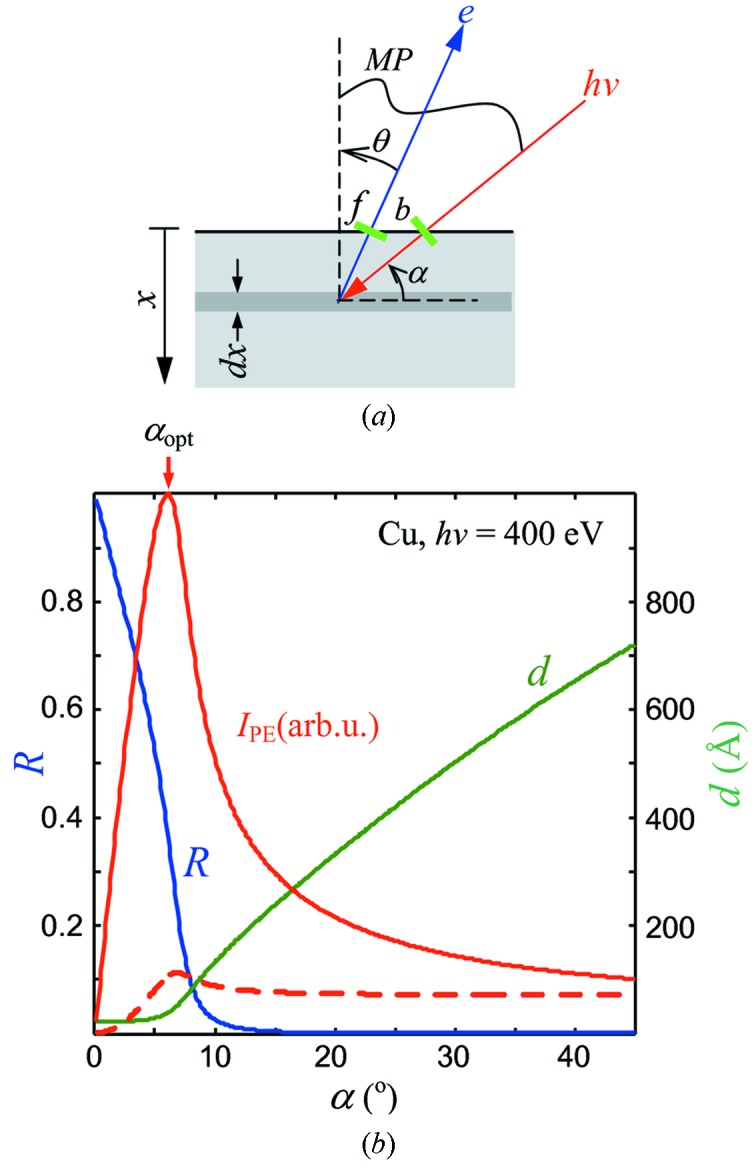
(*a*) Sketch of the photoelectron emission process. (*b*) Angle dependences of the X-ray reflectivity *R*, absorption depth *d* and the corresponding normal-emission *I*_PE_ for Cu at *h*ν = 400 eV in the FA (solid line) and FOVL (dashed) regimes. The decrease of *d* combined with an increase of *R* towards more grazing α forms the peak of *I*_PE_, prominent in the FA regime and only moderate in the FOVL regime.

We will first recap the basic formalism describing the X-ray excited photoelectron current 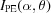 as a function of the X-ray grazing-incidence angle α and photoelectron emission angle 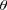 relative to the surface normal (see Fig. 1*a* ▶). According to the Beer–Lambert law, the electromagnetic field intensity *S*(*x*) in the media exponentially decreases with depth *x* as *S*(*x*) = 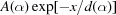, where *d*(α) is the electromagnetic field penetration depth (perpendicular to the surface) and 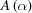 is a normalization coefficient. The latter is defined by the condition that 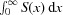, expressing the total absorption in the media, obeys the complementarity principle and is thus proportional to 1 − *R*, where *R* is the X-ray reflection coefficient. Performing this integral and equating it to 1 − *R*, we immediately obtain 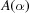 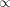 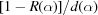 and
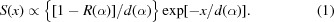
Then, neglecting the photoexcitation matrix elements and the photoelectron refraction important only at low energies, the photoelectron intensity 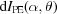 originating from a layer with a thickness d*x* placed at depth *x* is proportional to the power absorbed in the layer 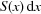 multiplied by the photoelectron transmission through the overlayer 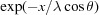, where λ is the photoelectron attenuation length (Powell *et al.*, 1999 ▶). Note that in contrast to *d*(α) taken perpendicular to the surface and having the meaning of depth, λ is taken along the photoelectron path and has the meaning of length. Integration of 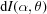 over the depth yields
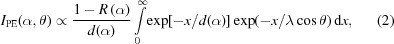
which evaluates to
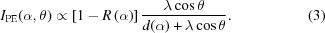



## 3. View factor   

The above formalism implied that the analyzer collected all photoelectrons emerging at the sample. Now, the above expression (3) should be multiplied by a geometrical view factor 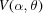 which is defined by the relation of the incident-beam cross section *b* and the analyzer FOV *f* (see Fig. 1*a* ▶) in their projections to the sample, 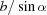 and 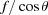, respectively. Obviously, with a less grazing α and more grazing 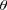, when the inequality 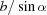 < 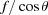 arises we have full photoelectron acceptance and 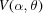 is identically equal to 1. In the opposite case the acceptance is FOV-limited and 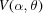 is equal to the ratio of the FOV and beam projections,
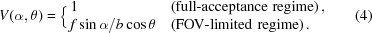
The full-acceptance (FA) regime implies that all photoelectrons emerging throughout the X-ray footprint on the sample are intercepted by the analyzer FOV; this is typical of the current synchrotron sources delivering a beam focused to some 10 µm and below. The FOV-limited (FOVL) regime (often referred to as the overfilled analyser slit) implies the loss of the photoelectrons outside the analyzer FOV; this is typical of the laboratory X-ray or older synchrotron sources with their spot being of the order of 1 mm. Our formalism for the FOVL regime is equivalent to that of Henke (1972 ▶) who back in 1972 implied exactly this situation. An illustrative comparison of the FA and FOVL regimes can be found, for example, at http://goliath.emt.inrs.ca/surfsci/arxps/introcss.html.

## 4. Numerical examples and analysis   

We will now use the above formalism in practical calculations. We restrict ourselves to the normal emission 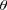 = 0. In this case the formulas (3)–(4) reduce to
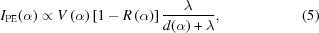
with 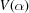 = 1 for the FA regime and 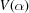 = 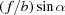 for the FOVL regime. The calculations were performed for the paradigm metal Cu at *h*ν = 400 eV. The numerical values of *R* and *d* were taken from the X-ray database readily availably on the Web (Henke *et al.*, 1993 ▶). The photoelectron energy was taken to be equal to *h*ν as relevant for the valence-band XPS. The corresponding λ was taken as the inelastic mean-free path^1^ and calculated according to the *TPP-2M* formula (Powell *et al.*, 1999 ▶) using the NIST Standard Reference Database implemented in the program *IMFPWIN* (NIST, 2011 ▶). The FOVL calculations assumed *f* = *b* and therefore 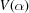 = 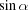.

Fig. 1(*b*) ▶ shows the *R*(α) and *d*(α) curves of the X-ray database values, as well as the *I*_PE_(α) curves calculated for the FA and FOVL regimes. We will now discuss the general trends seen in this figure.

(i) The region of less grazing α away from α_c_. Simple geometrical considerations give here *d*(α) ∝ sinα. Furthermore, *d*(α) >> λ and *R*(α) ≃ 0. This simplifies the formula (5) to 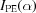 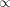 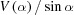. For the FA regime, 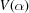 = 1 and the remaining 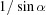 dependence reflects the gradual increase, when going to more grazing angles, of the power absorbed in the surface region where the photoelectrons are coming from. For the FOVL regime, the 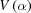 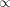 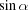 factor reflecting the analyzer FOV overfilling compensates this trend to constant 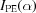, in agreement with Henke’s results (Henke, 1972 ▶). Therefore, the intensity gain with more grazing α can be achieved in this region only under the FA experimental conditions.

(ii) The region near α_c_. Here *d*(α) in (5) sharply reduces when going to more grazing angles to dramatically increase *I*_PE_. The counter-trend is the increase of *R* to reduce the total absorption and thus *I*_PE_. These opposite trends form the *I*_PE_ peak identifying the optimal incidence angle α_opt_. In the FA regime the intensity gain is dramatic. In the FOVL regime the factor 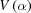 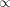 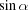 moderates the gain to a factor of ∼1.5, in agreement with the previous theoretical and experimental results (Henke, 1972 ▶; Kawai *et al.*, 1995 ▶), and slightly shifts α_opt_ to less grazing angles. In the following we will concentrate on the FA regime, as it is more effective and relevant for modern synchrotron instrumentation.

**Figure 2:**
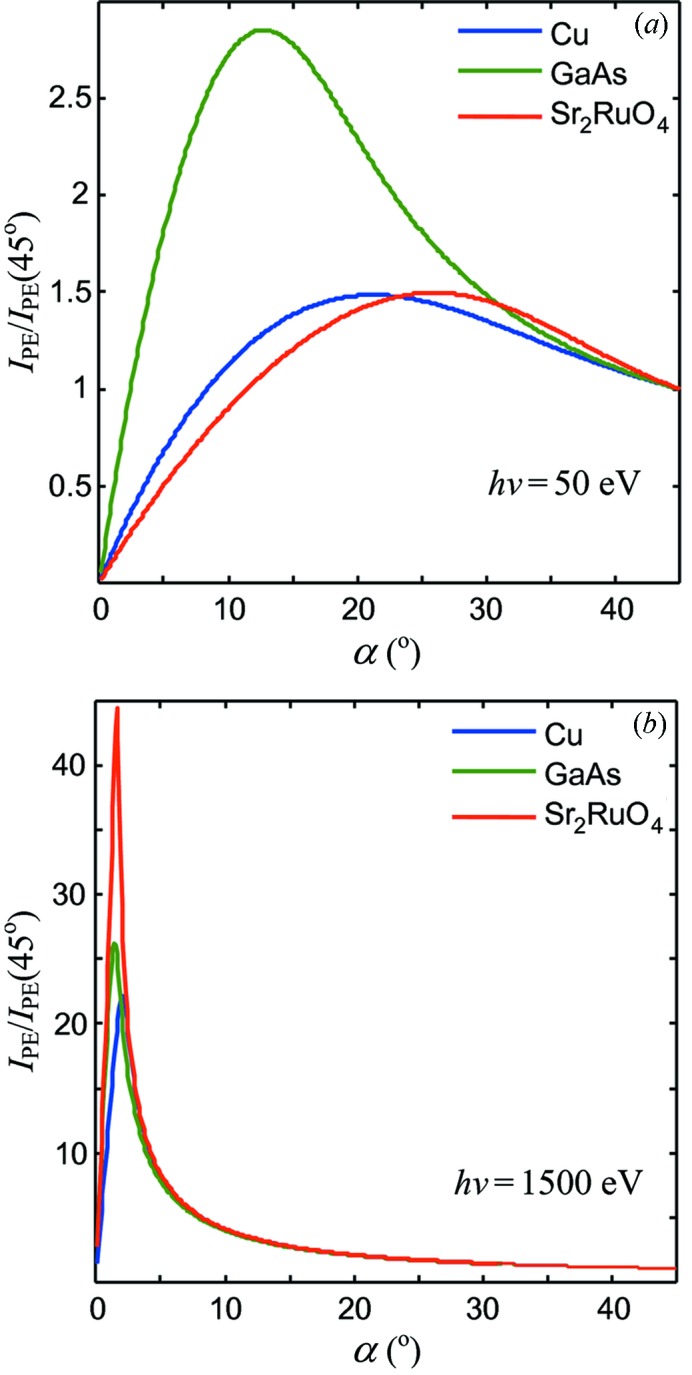
Dependences of the normal-emission *I*_PE_ on α (normalized to *I*_PE_ at 45°) at two photon energies in the FA regime for three paradigm materials. With increase of *h*ν the *I*_PE_ peak moves to more grazing α, sharpening and dramatically scaling up in amplitude.

To assess the universality of the above picture, the calculations were extended to another two paradigm materials, the semiconductor GaAs and strongly correlated material Sr_2_RuO_4_, and to two very different *h*ν values, 50 eV and 1500 eV. The results are shown in Fig. 2 ▶. As we have seen above, the *R*(α) and *d*(α) angular dependences combined with λ form the pronounced *I*_PE_ peak at α_opt_ near α_c_. In the low-energy case the peak appears at less grazing α; it is broad and less pronounced compared with *I*_PE_ at 45°. With increase of *h*ν the peak becomes more grazing, dramatically scales up in amplitude, and sharpens. These general trends appear to be fairly material-independent.

## 5. Optimal X-ray incidence angle   

The above calculations were extended to determine the α_opt_ angle maximizing *I*_PE_ (see Fig. 1*b* ▶) in a wide energy range from VUV photons of *h*ν = 50 eV to hard X-rays of 3.5 keV.

**Figure 3:**
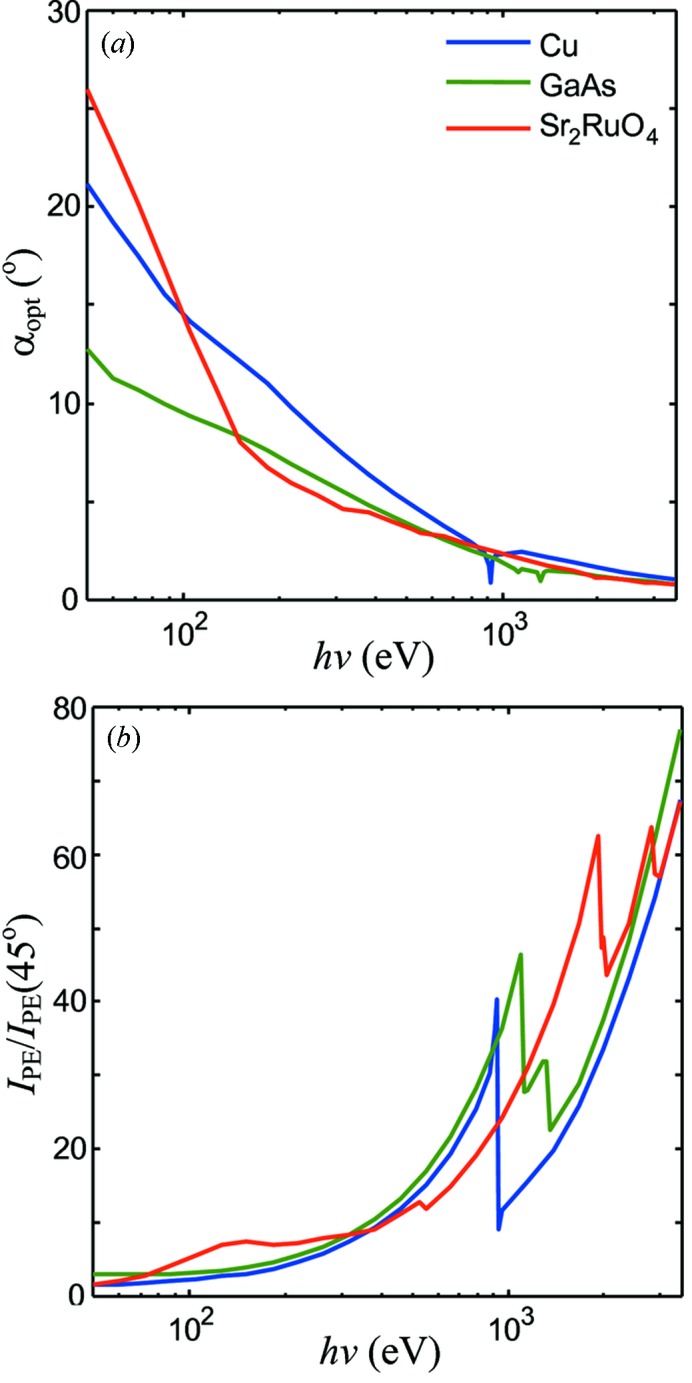
(*a*) Energy dependences of the α_opt_ optimal angle. (*b*) Corresponding *I*_PE_ intensity gain (normalized to *I*_PE_ at 45°) in the FA regime. α_opt_ becomes more grazing with increasing energy, accompanied by a dramatic increase of the *I*_PE_ gain.

**Figure 4:**
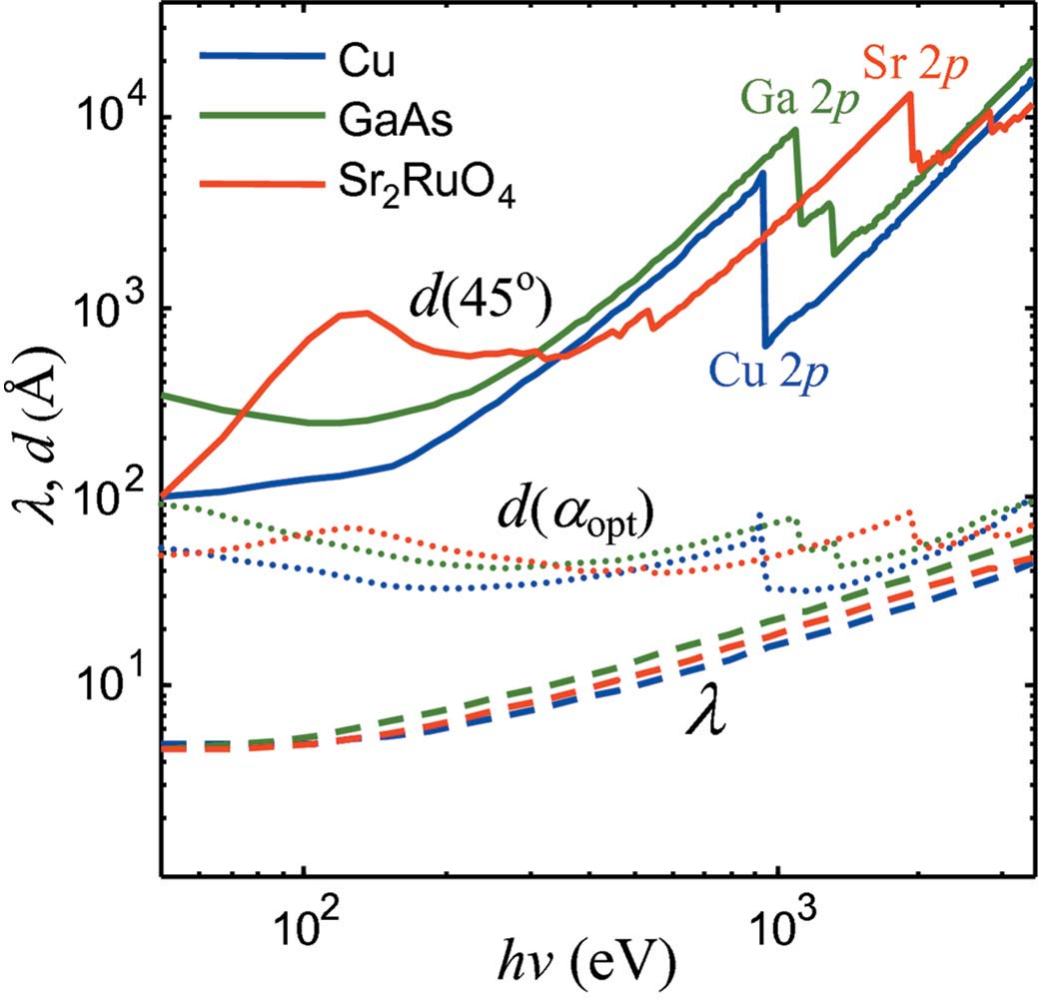
Energy dependences of *d* at α = 45° (solid lines) compared with λ (dashed lines). The penetration of X-rays increasing with energy faster than that of photoelectrons forces the decrease of α_opt_ in Fig. 3 ▶. Also shown is *d* at the energy-dependent α_opt_ (dotted lines).

Fig. 3(*a*) ▶ shows the central result of our evaluation: the calculated energy dependences of α_opt_ for our three paradigm materials. The dependences drop from less grazing values of the order of 20° at the 50 eV end to very grazing values of about 1° at the 3.5 keV end. The physics of this behavior becomes clear from Fig. 4 ▶, which presents the *h*ν dependences of *d* taken at α = 45°, far away from α_c_ (note its sharp drop at the transition-metal 2*p* absorption edges), compared with the energy dependences of λ. The increase of *d* through the shown energy range is about an order of magnitude stronger than that of λ. The need to concentrate the absorbed X-ray power in a better balance with λ forces α_opt_ to become more grazing with *h*ν. Working in the same direction is also the evolution of *R*(α), whose onset shifts with *h*ν towards more grazing angles. Returning to Fig. 3(*b*) ▶, we also note that the intensity gain achieved at α_opt_ increases dramatically with *h*ν.

Fig. 4 ▶ also shows the *h*ν dependences of *d* at α_opt_. Due to α_opt_ becoming progressively more grazing, *d*(α_opt_) flattens compared with *d* at 45°. It is interesting to note that, somewhat counterintuitively, the normal-emission *I*_PE_ maximum at α_opt_ in general does not balance *d* and λ. This manifests the modulating effect of *R*(α) which reduces the absorbed X-ray power towards more grazing α. The balance between *d* and λ improves, however, at the high-energy end. In this case *d* starts to affect the probing depth of the XPS experiment on equal footing with λ.

## 6. Practical considerations   

The above gain in *I*_PE_ of a few tens of times is huge. However, it requires extreme grazing angles, and can only be fully realised in the FA regime, *i.e.* while the analyser FOV intercepts a whole light spot that blows up proportionally to (sinα)^−1^. Below we give some simple considerations to maintain or at least stay close to this regime at grazing angles.

(i) The experimental geometry of the synchrotron-based XPS facilities should take into account the elliptical cross section of the incident beam with its relatively small vertical size *b*_V_ and large horizontal size *b*_H_. This means that the sample should be taken to grazing incidence by rotation around the horizontal axis to increase the smaller *b*_V_ rather than the larger *b*_H_. In this case the measurement plane (MP) formed by the incident beam and analyzer lens axis (see Fig. 1*a* ▶) is vertical. This geometry has been implemented, for example, at the highly efficient soft X-ray ARPES facility at the ADRESS beamline (Strocov *et al.*, 2010 ▶) of the Swiss Light Source.

(ii) The analyzer FOV is determined by the operation mode of the analyzer lens (Mårtensson *et al.*, 1994 ▶; Wannberg, 2009 ▶) and on the opening and orientation of the analyzer slit. For the magnification (essentially imaging) modes the FOV is just the slit dimension *s* divided by the lens magnification *M*, *f* = *s*/*M*. Obviously operation at grazing angles will benefit from low magnifications. Furthermore, the analyzer slit should be oriented in the MP, because in this case *s* will be determined by the relatively large slit length (usually around 20 mm) compared with its relatively small width (around 200 µm). With the modern synchrotron sources and vertical MP geometry, the FA regime is in this case practically unlimited in grazing angles. The transmission lens modes normally deliver even larger FOVs compared with the magnification modes, but its determination does not obey the simple imaging considerations because the photoelectrons collected at the same point at the slit can originate from different points on the sample. The most restrictive on the spot size are the angle-resolving (ARPES) operation modes, because the best angular resolution is ensured within a FOV of the order of only 100 µm.

(iii) The XPS analyzers are normally mounted fixed at one of the flanges of the vacuum chamber. The angle between the analyzer lens and the incident light therefore stays fixed. In this case it is reasonable to optimize α near the low-energy end of the required *h*ν range because at higher energies α will sit on the more gradual right-hand side of the *I*_PE_ peak (see Fig. 2 ▶). With the soft X-ray energy range starting at ∼300 eV, this yields α_opt_ ≃ 8° (see Fig. 3*a* ▶). For the ARPES measurements with *f* ≃ 100 µm, the FA regime will then require *b*_V_ = *f*/sinα_opt_ ≃ 14 µm, which is hardly a problem for current synchrotron instrumentation. With the hard-X-ray energy range starting at ∼2.5 keV, we arrive at α_opt_ ≃ 1.3°. For the ARPES measurements in this region (see, for example, Gray *et al.*, 2011 ▶) the FA regime will be limited by *b*_V_ ≃ 2.3 µm, which already requires aggressive focusing of the incident beam.

We also note that an additional advantage of a grazing α is a reduction of the inelastic secondary electron background, because the concomitant decrease of *d* reduces the secondary electron background originating from photoelectrons excited in the sample depth beyond λ and inelastically scattered on their way to the surface (see, for example, Kawai *et al.*, 1995 ▶).

## 7. Conclusion   

We have analysed the interplay between the X-ray reflectivity, X-ray absorption depth and the photoelectron attenuation length in the photoelectron emission process. With increase of energy from the VUV to hard X-rays, the optimal X-ray incidence angle α_opt_ delivering maximal XPS signal becomes progressively more grazing, from a few tens of degrees to about 1°. This is accompanied by an intensity gain at α_opt_, increasing from insignificant to a factor of a few tens as long as the experiment stays in the FA regime with the whole X-ray footprint on the sample intercepted within the analyzer FOV. These trends are fairly material-independent. The practical utilization of the intensity gain at α_opt_ by the (synchrotron-based) XPS spectrometers in general requires a vertical measurement plane with in-plane analyzer slit orientation and, particularly for ARPES experiments towards the hard X-ray energies, focusing of the incident X-ray beam down to a few micrometers.

## Footnotes

1 We note that the electron attenuation length in the crystalline media is in general limited only by the inelastic scattering. The picture of elastic scattering appearing in the Boltzmann transport equation is superseded by the band theory where the Bloch electrons are formed by self-consistent multiple elastic scattering on the periodic potential and thereby propagate through the crystal without attenuation. The elastic attenuation is relevant in this theory only for the evanescent Bloch waves in the band gaps important at low energies (Barrett *et al.*, 2005 ▶, and references therein).
